# Long term impact of the WHI studies on information-seeking and decision-making in menopause symptoms management: a longitudinal analysis of questions to a medicines call centre

**DOI:** 10.1186/s12905-021-01478-z

**Published:** 2021-10-04

**Authors:** Rifani B. Natari, Samantha A. Hollingworth, Alexandra M. Clavarino, Kaeleen D. Dingle, Treasure M. McGuire

**Affiliations:** 1 School of Pharmacy, The University of Queensland, 20 Cornwall Street, Woolloongabba, QLD 4102 Australia; 2 Department of Pharmacy, Jambi Regional Psychiatric Hospital, Jambi, 36129 Indonesia; 3 School of Public Health, University of Queensland, Herston, QLD 4006 Australia; 4 School of Public Health and Social Work, Queensland University of Technology, Kelvin Grove, QLD 4059 Australia; 5 Mater Pharmacy, Mater Health, Brisbane, QLD 4101 Australia; 6 Faculty of Health Sciences and Medicine, Bond University, Robina, QLD 4226 Australia

**Keywords:** Menopause, Estrogen replacement therapy, Information seeking behaviour, Decision making, Drug information services

## Abstract

**Background:**

While women are taking a greater role in decisions about menopause symptom management, the legacy of the Women’s Health Initiative (WHI) studies persist. Despite hormone therapy (HT) being effective in reducing all-cause mortality, many women seeking relief of menopausal symptoms exaggerate HT harms and overstate the perceived benefits or ignore the risks of alternative therapies. We aimed to explore the longitudinal impact of the widely-publicised WHI 2002 study on women’s information-seeking and describe determinants of decision-making about managing menopausal symptoms.

**Methods:**

In a longitudinal analysis of both quantitative and qualitative data, we explored consumer questions about menopause-related medicines received by two Australian medicines call centres (1996–2010) before, during, and after WHI 2002. We analysed calls by age and gender of caller and patient, their relationship, postcode, enquiry type, and motivation to help-seek. We compared calls regarding HT and herbal medicines (HM) with the rest of calls, and thematically analysed question narratives across the three time-periods.

**Results:**

There were 1,829 menopause-related calls received of over this time-period, with a call surge, primarily from women in their mid-fifties, in the two months after the WHI 2002 publication. Two in three calls were motivated by negative media reports as women sought support for decision-making, primarily reassurance to cease HT. While HT safety concerns persisted for eight years post-publication, the nature of information-seeking changed over time. Callers subsequently sought reassurance to use menopause treatments together with their other medicines; and pursued HT substitutes, including HM, in response to HT product discontinuation.

**Conclusions:**

Women sought information or reassurance to support a decision, based on dynamic changes in internal (symptom or risk intolerance, attitude towards menopause and treatment preferences) and external factors (perceived source trust and changes in treatment availability). In assessing HT benefit versus risk, women tend to overestimate risk with HT safety concerns persisting over time. Decision-making in managing menopause symptoms is complex and dynamic. Reassurance to reach or justify decisions from a perceived trusted source can support informed decision-making.

**Supplementary Information:**

The online version contains supplementary material available at 10.1186/s12905-021-01478-z.

## Background

Menopause is a natural and unique experience for every woman [1]; some easily pass through, while others have a difficult transition [1]. Vasomotor symptoms (VMS) are most common during the transition (prevalence 30–70%) [1] and are associated with depression [2], lower health-related quality of life [3, 4], socio-economic factors, and increased use of healthcare services [4].

Many were uncertain about how best to manage symptoms after the publication of the Women’s Health Initiative (WHI) studies in 2002 and 2004 [5, 6]. These studies tested the heart-protective effect of hormone therapy (HT) in women who were older (50–79 years) than those in earlier studies (45–64 years) [7, 8]. WHI 2002 was prematurely terminated after HT use by women with an intact uterus was unexpectedly associated with an increased risk of breast cancer, with no apparent beneficial cardiovascular effects [5]. Widespread negative media coverage led to a substantial and rapid decrease in HT use worldwide [9]. Many women ceased HT abruptly, with some recommencing HT due to intolerable symptoms [10]. A second WHI study of estrogen therapy (ET) in women with a hysterectomy was also prematurely terminated due to an increased risk of stroke with no increase in breast cancer risk [6] yet these conflicting findings were largely ignored by the media. Subsequent reanalysis found that both studies were confounded by age [11] with a ‘window of opportunity’ for cardiovascular health benefits if HT was initiated before 60 years or within ten years of menopause [11]. The 2016 Revised Global Consensus Statement on Menopausal Hormone Therapy [12] reaffirmed that HT remains the most effective treatment for vasomotor symptoms and significantly lowers the risk of osteoporosis-related fractures in postmenopausal women. HT is effective in vulvovaginal atrophy and may improve sexual function and other related symptoms such as joint and muscle pains, mood changes and sleep disturbances. However, prescriptions for HT have continued to tumble. Unfortunately, the WHI 2002 findings in older women with an intact uterus were generalised to all forms of HT, including estrogen alone in younger hysterectomised women. Prescriptions for HT declined steeply, with estimates that within 18 months of WHI 2002, half of US women using systemic HT stopped treatment. [13] Sarrel and colleagues [14] used WHI 2004 data [15] indicating a higher rate of mortality among hysterectomised women aged 50 to 59 years assigned to placebo than estrogen over a 10-year follow-up, to determine how this rate of excess mortality translated into an aggregate toll of premature death through estrogen avoidance at the population level (2002–2011). Their analysis concerningly suggested that between 18,601 and 91,610 excess deaths occurred among hysterectomised women aged 50–59 years following the publication of the original WHI.

Although some women continue to use HT [16], as an alternative to HT, many women also use herbal medicines (HM) [17] antidepressants [17], GABAergic agents [17], clonidine [17], and non-pharmacological approaches for VMS [18]. The changing HT evidence and multiple treatment options (including no treatment) have made decision-making more complex [19, 20].

Women are taking a greater role in decisions about managing symptoms [21]. The consumer’s role seems to dominate the clinician’s when deciding on preferred treatment [21] but professional guidance is still needed and often sought when evidence of efficacy, safety, and multiple treatment options add to confusion [19, 20]. However, the legacy of the WHI studies persists. Despite hormone therapy being effective in reducing all-cause mortality [22], many women seeking relief of menopausal symptoms exaggerate HT harms, overstate the perceived benefits of alternative therapies, or ignore their risks [23–25].

Women’s decision-making processes in managing menopause have been modelled in normal [19] and surgical menopause [20]. Existing models suggest decision-making is driven by both internal (e.g. individual characteristics, values, attitudes, beliefs and preferences) and external factors (e.g. healthcare provider context, facts and information) [19, 20] but these models have limitations. Evidence was collated mainly from cross-sectional studies, with many conducted pre-WHI 2002 [19, 20]. There are comparatively few recent studies focusing on women in the active process of decision-making [24, 26].

Women seek information to manage their health from a range of sources including social networks, social media, and the internet [9, 27]. Some women may be uncomfortable using the internet, preferring immediate feedback or an opportunity to ask questions and discuss potentially opposing ideas [28, 29]. Where consultation time is limited, others might be reluctant to be perceived as ignorant or question their clinician’s judgement [30]. A consumer medicines call centre (MCC) operated by pharmacists provides an alternative, anonymous source for obtaining reliable and evidence-based medicines information [31].

We sought to understand the information-seeking and decision-making behaviour of women accessing a MCC with menopause symptom management concerns, as evidence changed over time. We aimed to: (1) longitudinally explore the impact of WHI 2002 on women’s information-seeking behaviours (2) compare information-seeking for HT and menopause-related HM, and (3) identify factors affecting decision-making for menopause symptoms.

## Methods

### Data sources

**Fig. 1:**
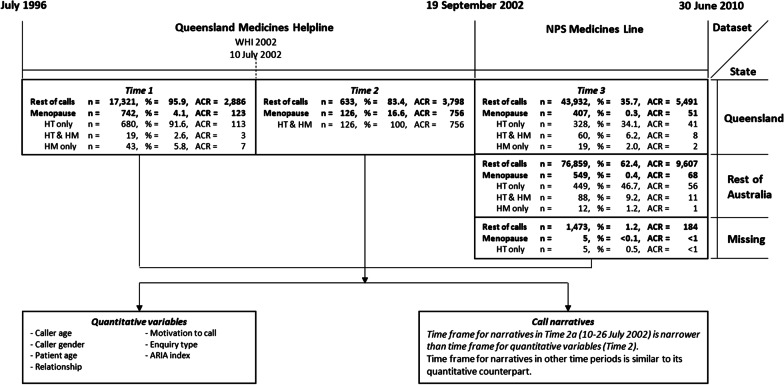
Flow chart describing the datasets explored in the study; critical time-points; data subset by state and medicine type-related calls, and; quantitative variables and call narratives obtained for analysis. WHI = Women’s Health Initiative study, HT = hormone therapy, HM = herbal medicines, ACR = annualised call rate

We longitudinally analysed routinely-collected health service data from two Australian MCCs operated by clinical pharmacists from Mater Health, Brisbane: (1) Queensland Medication Helpline (QMH), a state-wide service operating from July 1996 to September 2002, i.e. pre-WHI and two months post-WHI 2002; and (2) NPS MedicineWise, formerly the National Prescribing Service (NPS) *Medicines Line* (ML), a nationwide service, September 2002–June 2010. Together these datasets provide continuous, longitudinal data over 14 years; and can be divided into three time periods (Fig. 1):

- *Time 1* Pre-WHI 2002 (June 1996–9 July 2002), menopause-related calls were only 4.1% of all calls, most related to HT only.
- *Time 2* WHI 2002 publication and associated negative media (10 July–19 September 2002), menopause-related calls increased to 16.6% of all calls.
- *Time 3*: post-WHI (20 September 2002–30 June 2010), menopause-related calls decreased to 0.3% of Queensland and 0.4% of calls from the rest of Australia.

We quantitatively and qualitatively analysed these data. The time ranges were identical for *Time 1* and *Time 3;* but we used a deliberately narrower ‘window’ (10–26 July 2002, *Time 2a*) for the qualitative analysis (Fig. 1) to capture questions and concerns in the period immediately following WHI 2002 and the media response. During these 13 service days, QMH pharmacists kept detailed information on callers’ menopause-related questions and concerns—some with overwhelming anxiety—and any actions prior to service contact.

We conducted and reported this research in accordance with the REporting of studies Conducted using Observational Routinely-collected health Data (RECORD) guideline [32]. We had ethical approval from the Mater Health Services Human Research Ethics Committee (HREC/13/MHS/80).

### Data collection

Call data were recorded manually on a scannable collection form and transformed into an electronic dataset. Most variables were consistently recorded over time, including caller and patient demographic characteristics, question enquiry type, call motivation, medicines involved, the question, and answer. Question narratives were only electronically recorded in selected periods due to limited funding but we hand-searched forms for additional narratives across the study period.

### Data classification

Medicines of interest were mapped to the Anatomical Therapeutic Chemical code: [33] G03CA (estrogens), GO3F (estrogen plus progestogen therapy, EPT), G03CX01 (tibolone), and G03BA03 (testosterone). We filtered data from female consumers by age to include calls from women who might experience premature menopause: ≥ 35 years for women calling for estrogen therapy and ≥ 48 years for EPT; calls were considered’rest of calls’ (ROC) if patient age was under the cut-off. We filtered the testosterone calls by patient gender (‘female’ or ‘blank’) to enhance call retrieval and we excluded non-menopause data. We examined calls for five HM: black cohosh, wild yam, soy-based products, chaste tree, and red clover.

## Variables of interest

We extracted data on variables: gender and age of the caller and patient, caller-patient relationship (self, partner, parent, child, other family, friend and client), enquiry type, call motivation, and postcode. Pharmacists classified the enquiry type and call motivation into predetermined categories [31]. We collapsed the 21 enquiry types into seven groups: (1) side effects, (2) risk/benefit, (3) pragmatics of use (e.g. administration, dose, withdrawal and efficacy, and stability/storage/disposal), (4) interactions, (5) mechanism/profile (e.g. indication, identification, formulation), (6) treatment/prophylaxis, and (7) logistics and miscellaneous (e.g. availability, comparison, cost, generic, subsidised medicines). The 17 call motivations were collapsed into six groups: (1) inadequate information, (2) second opinion, (3) worrying symptoms, (4) conflicting information, (5) the media and (6) other. We mapped caller postcode to the Accessibility/Remoteness Index of Australia (ARIA), which measures the remoteness of areas from service centers [34, 35]. We compared proportions of calls from each ARIA with population data to generate relative call frequencies expressed as a ratio [34, 35].

### Analysis

#### Quantitative analysis

We compared menopause-related calls and ROC over time and by medicines class (HT and HM). Some data (QMH) were only from Queensland so we assessed whether call characteristics differed between Queensland and other states in the Australian (ML) dataset and between Queensland callers of QMH and ML, respectively. We used a one-way analysis of variance (ANOVA) and Pearson’s chi-square test to test for any differences. We conducted *post-hoc* analyses, including Tukey’s honesty significant test and standardised residuals method [36], to further explore any differences among categories. We applied the Bonferroni correction to a p-value of 0.05 to avoid false-positive results [36]. We used Microsoft Excel 365 (Microsoft, Redmond, WA, USA) and SPSS (version 25.0, IBM Corp, Armonk, NY, USA).

#### Qualitative narratives

The purpose of the qualitative data analysis was to provide further insight into the results of our quantitative analyses. All question narratives were collected and analysed thematically according to the results identified in our quantitative analyses. We used a system of coding, comparison, and clustering with a basic coding frame developed from our quantitative results [37]. To enhance the rigour of this analysis two coders separately coded each question narrative. Any discrepancies or revisions to coding were resolved through consensus.

## Results

### Impact of the WHI 2002 publication on call characteristics

Most menopause-related calls were made by women in their mid-50s (average 56.5–59.3 years) from areas classified as either highly accessible or accessible, and seeking help for themselves (Table 1). Call characteristics were compared among: (1) menopause-related and ROC over time; (2) Queensland and other states in the Australian dataset; and (3) Queensland callers of QMH and ML (Additional file 1). The differences in key call characteristics between menopause and ROC were consistent and stable over time. There were no differences in menopause-related calls for caller gender, relationship between caller and patient, nor ARIA when we compared calls originating from Queensland vs other states/territories; or over time (Additional file 1).

**Table 1:** Comparison of characteristics of menopause-related calls across three time periods

Characteristics	*QMH*		*ML*	p-value	Note
	*Time 1*	*Time 2*	*Time 3*		
	(n = 742)	(n = 126)	(n = 961)		
*Callers age, years*				0.004	Comparison, ΔMean (95% CI)
Mean (SD)	56.5 (9.6)	59.3 (8.5)	57.7 (10.5)		*Time 1* vs. *Time 2*: -2.80 (-5.07, -0.54) *Time 1* vs. *Time 3*: -1.20 (-2.35, -0.05)
*Callers gender, %*				0.611	
Male	4.0	4.0	3.2		
Female	94.2	95.2	96.8		
*Patients age, years*				0.008	Comparison, ΔMean (95% CI)
Mean (SD)	57.1 (9.7)	59.9 (8.9)	58.0 (10.2)		*Time 1* vs. *Time 2*: -2.80 (-5.04, -0.56)
*Relationship of caller, %*				0.006^a^	
Self	89.1	88.9	95.1		
Partner	2.2	4.0	1.9		
Client (carer/patient) ^a^	2.0	-	1.7		
Child/friend/other family ^a^	2.8	1.6	0.8		
Parent ^a^	1.2	0.8	0.5		
*Call motivation, %*				< 0.001	
Inadequate information ^b^	14.2	9.5	38.5		
Second opinion	14.7	8.7	24.5		
Worrying symptom	22.2	4.8	19.7		
Conflicting information	7.8	0.8	8.8		
Other	13.5	1.6	6.8		
Media ^b^	7.4	66.7	1.3		
*Enquiry type, %*^c^				< 0.001	
Side effects ^d^	24.8	15.9	23.2		
Pragmatics of use	16.2	14.1	11.8		
Treatment/prophylaxis	13.4	8.2	8.5		
Risk/benefit ^b,d^	12.7	46.5	17.4		
Logistics and miscellaneous	12.7	8.2	9.4		
Mechanism/profile	12.5	4.7	14.4		
Interaction ^b,d^	7.6	2.4	15.1		
Enquiry type—safety	45.1	64.8	55.7		
*ARIA index, relative call frequency*^e^				0.209	
Highly accessible	1.14	1.17	1.22		
Accessible	0.55	0.46	0.48		
Moderately accessible	0.48	0.44	0.32		
Remote	0.42	-	0.53		
Very remote	0.56	0.80	1.00		

Comparison between group in callers’ and patients’ age was obtained from Tukey’s *post-hoc* analysis. Only comparison with statistically a significant result at the p ≤ 0.05 is presented

QMH = Queensland Medication Helpline, ML = National Prescribing Service Medicines Line, Time 1 = pre-the Women’s Health Initiative (WHI) 2002 study (1996 – 9 July 2002), Time 2 = post-WHI 2002 (10 July – 19 September 2002), Time 3 = QMH = Queensland Medication Helpline, ML = National Prescribing Service Medicines Line, Time 1 = pre-the Women’s Health Initiative (WHI) 2002 study (1996 – 9 July 2002), Time 2 = post-WHI 2002 (10 July – 19 September 2002), Time 3 = QMH = Queensland Medication Helpline, ML = National Prescribing Service Medicines Line, Time 1 = pre-the Women’s Health Initiative (WHI) 2002 study (1996 – 9 July 2002), Time 2 = post-WHI 2002 (10 July – 19 September 2002), Time 3 = *S*eptember 2002 – June 2010, SD = standard deviation, ΔMean = mean difference between two groups, CI = confidence interval, ARIA = Accessibility Remoteness Index of Australia

^a^Value of client (carer/patient), child/friend/other family, and parent were combined to meet the assumption of Pearson’s chi-square test, i.e. less than 20% of cells have an expected value less than 5

^b^Denotes the category with column proportions that differ significantly across time-periods at the p ≤ 0.05 with Bonferroni correction

^c^Some QMH calls have more than one question. Proportion in this variable reflects the proportion of the number of enquiry types to total enquiry type (Time 1, number (n) menopause questions = 1,739; Time 2, (n) menopause = 170)

^d^All categories related to safety in Enquiry type

^e^Relative call frequency was calculated by dividing the proportion of calls by proportion of population in the specific ARIA index

Menopause-related calls surged in the two months after WHI 2002 (July 2002, Fig. 1); two in three calls (66.7%) were motivated by media reports. Prior to WHI 2002, women were motivated to ring because of worrying symptoms and inadequate information (Table 1). The media effect was relatively short-lived, with only 13 questions (~ 2 calls/year) prompted by media after September 2002. Information gaps or concerns about medication safety were the primary reasons for calling, irrespective of time. Safety enquiries ranged from 45 to 65%, with the highest frequency immediately after WHI 2002 largely due to an increase in queries about HT’s risks versus benefits (Table 1).

Calls about menopause-related HM were few: only 8.4% before WHI 2002 and 18.6% later on (Fig. 1). Only two of 126 callers asked a question about HM at immediately after WHI 2002 when HT concerns predominated. After September 2002, one in three (34.7%) HM calls concerned interactions (Table 2).

**Table 2:** Comparison of call characteristics between menopause-related calls about hormone therapy and herbal medicines across three time periods

Characteristics	*Queensland Medication Helpline*				*NPS Medicines Line*		
	*Time 1*			*Time 2*	*Time 3*		
	HT only (n = 680)	HT and HM (n = 19)	HM only (n = 43)	HT and HM^a^ (n = 126)	HT only (n = 782)	HT and HM (n = 31)	HM only (n = 148)
*Caller age, years*							
Mean (SD)	56.7 (9.8)	53.3 (7.9)	56.6 (5.8)	59.5 (8.5)	58.6 (10.6)	53.3 (9.4)	56.0 (5.8)
*Caller gender, %*							
Male	3.8	-	9.3	4.0	3.3	3.2	2.7
Female	94.4	100.0	88.4	95.2	96.7	96.8	97.3
*Patients age, years*							
Mean (SD)	57.4 (9.9)	53.1 (7.5)	56.3 (5.8)	59.9 (8.9)	58.8 (10.5)	54.0 (8.7)	55.9 (5.8)
*Relationship of caller, %*							
Self	89.1	94.7	86.0	88.9	95.8	96.8	91.2
Partner	2.1	-	4.7	4.0	1.8	3.2	2.0
Client (carer/patient)	1.9	5.3	2.3	-	1.3	-	4.1
Child/friend/other family	2.6	-	7.0	1.6	0.8	-	1.4
Parent	1.3	-	-	0.8	0.4	-	1.4
*Call motivation, %*							
Inadequate information	13.7	10.5	23.3	9.5	37.2	41.9	44.6
Second opinion	14.6	21.1	14.0	8.7	24.4	25.8	24.3
Worrying symptom	23.5	5.3	9.3	4.8	21.2	19.4	11.5
Conflicting information	7.9	5.3	7.0	0.8	9.3	-	8.1
Other	13.1	26.3	11.6	1.6	6.4	12.9	7.4
Media	6.9	10.5	14.0	66.7	1.0	-	3.4
*Enquiry Type, %*^b^							
Side effects	25.8	17.1	17.5	16.2	25.8	19.4	18.2
Pragmatics of use	15.6	20.9	22.2	13.8	15.6	9.7	14.2
Treatment/prophylaxis	13.8	9.3	11.1	8.4	13.8	12.9	6.8
Risk/benefit	12.4	14.0	17.5	46.7	12.4	19.4	16.9
Logistics and miscellaneous	13.1	8.5	9.5	8.4	13.1	12.9	0.7
Mechanism/profile	11.9	17.8	17.5	4.2	11.9	9.7	8.1
Interaction	7.4	12.4	4.8	2.4	7.4	12.9	34.5
*ARIA index, relative call frequency*^c^							
Highly accessible	1.13	1.08	1.19	1.16	1.23	1.23	1.17
Accessible	0.53	0.83	0.38	0.46	0.42	0.16	0.81
Moderately accessible	0.51	0.21	-	0.44	0.31	1.08	0.30
Remote	0.43	-	1.77	-	1.00	-	-
Very remote	0.45	1.15	-	0.40	0.90	-	-

*Time 1* = pre-the Women’s Health Initiative (WHI) 2002 study (1996 – 9 July 2002), *Time 2* = post-WHI 2002 (10 July – 19 September 2002), *Time 3* = September 2002 – June 2010, HT = hormone therapy, HM = herbal medicine, SD = standard deviation, ARIA = Accessibility Remoteness Index of Australia

^A^only two calls asking about hormone therapy and herbal medicines in *Time 2*

^b^Some calls from Queensland Medication Helpline have more than one question recorded. The proportion in this variable reflects the proportion of the number of enquiry type to total enquiry type (*Time 1*, number of questions (n) HT only = 1,547, HT and HM = 63, HM only = 129; *Time 2*, n HT and HM = 170)

^c^Relative call frequency was calculated by dividing the proportion of calls by the proportion of population in the specific ARIA index

### Menopause narratives

We identified four primary themes (*safety concerns, pragmatics of use, therapy options,* and *regimen comparisons*) and 17 sub-themes from 749 menopause-related calls. There were 846 total questions (some narratives had more than one question): 229 from before WHI (*Time 1)*, 70 immediately after WHI (*Time 2a)*, and 547 from September 2002 (*Time 3)*. The nature of calls varied across the periods, but *safety concerns* predominated (59.4% *Time 1*, 98.6% *Time 2a*, 65.3% *Time 3),* with women tending to overestimate HT risk (Table 3). Immediately after WHI 2002 negative media coverage (*Time 2a*), one in three (32%) callers (32%) were particularly concerned about breast cancer. Many had abruptly ceased, or were seeking reassurance to cease, their HT (including women who had had a hysterectomy) because of breast cancer fears.

48-year-old woman - four years since she had a breast cancer removed.

“I’m really worried about taking estrogen in case it reactivates my breast cancer, but I just want to treat my terrible tiredness and hot feelings. What should I do?”

**Table 3:** Selected caller quotes for each of the themes and sub-themes identified across the three specified time periods

	*Time 1* – QMH Pre-WHI 2002 (Jun 1996–9 Jul 2002)	*Time 2a* –– QMH Immediately post WHI 2002 publication (10 – 26 July 2002) extracted from *Time 2*	*Time 3* – NPS *Medicines Line* (Sep 2002 – Jun 2010)
Total cases	742	126	961
Cases with narratives (Q)	165	70	514
Cases with > 1 theme	229	-	547
Denominator	229	70	547
*Themes*			
1. Safety concerns	n = 136 (59.4%)	n = 69 (98.6%)	n = 357 (65.3%)
Risk concern – breast cancer	Nil	22 (31.4%) 54-year-old (yo) woman *“I stopped my HRT yesterday after I read about it causing breast cancer. Today I feel really awful. – what should I do?”* 59 yo woman *“I have been using HRT for years but just found out about it being linked to breast cancer. I stopped it straight away; but is it too late? I’m so worried as my sister is already battling breast cancer.”*	Nil
Reassurance to withdraw (any safety concern)	12 (5.2%) 60 yo woman *“I had a hysterectomy and have been on Provera® (medroxyprogesterone) and Premarin® (conjugated estrogens) for three years. My doctor says the drugs aren’t prescribed any more. Why not prescribed now? Should I wean?*	Nil	26 (4.7%) 50 yo woman *“Can she stop taking Livial® (tibolone) abruptly after being on it for seven years?”*
Risk concern –in women who had a hysterectomy	5 (2.2%) 50 yo woman *“I have been on Provera® 5 mg (medroxyprogesterone) and Premarin® 0.625 mg (conjugated estrogens) for several months for menopausal symptoms. I feel much better but concerned about HRT and breast cancer story. I have no family history of breast cancer. Is there a risk of breast cancer with HRT use?”*	6 (8.6%) 63 yo woman *“I have had a hysterectomy, do these reports apply to me? What should I do? Can I stay on my HRT?”*	27 (4.9%) 62 yo women *“I had hysterectomy and has been used HRT since many years ago. I used Premarin® (conjugated estrogens) 0.3 cut in half due to 'scare in America'. I ran out the medicine and doctor in Kenmore clinic prescribes me Livial. I don’t know if Livial tablet, is it HRT therapy? Is it for osteoporosis?”*
Risk /safety concerns specified and unspecified symptoms mentioned	5 (2.2%) 52 yo woman On tamoxifen for treatment of breast cancer *“I am going ‘nuts’ with symptoms of vaginal dryness but my doctor said I just has to put up with it! What can I try instead?”*	2 (2.9%) 60 yo male ringing about wife who died of heart failure at 59 yo *“My wife was taking HRT and she died of heart failure. Did she die because of her HRT?”* Example of a ‘general concern’ 68 yo woman *“I have been using Ovestin vaginal cream as HRT. After hearing about the danger of HRT on the radio I’m really worried. Do these HRT risks apply to my Ovestin?”*	11 (2.0%) Symptoms described due to hormone imbalance (n = 184) e.g. insomnia, VMS, headache, depression, hirsutism 51 yo woman *“Can any of my medicines cause migraines: HRT, Livial® (tibolone)?”* Misc (non-hormonal) e.g. rash 62 yo woman *“Can I having an allergic reaction to Ovestin® (estriol) if I have a prickly feeling on my labia?”*
Clarifying/quantifying risk	Nil	Nil	5 (0.9%) 71 yo woman *“How much risk is there for me to get breast cancer or dementia when using Ovestin® (estriol) vaginal cream?”*
Clarification of symptom cause	46 (20.1%) 56 yo woman *“I have been on HRT for a while. My blood pressure was 170/100 3 weeks ago, and my last measurement was 150/100. I don’t experience any headaches. Can my HRT cause hypertension?”*	Nil	89 (16.3%) 56 yo woman *“Can Premarin® (conjugated estrogens) cause agitation, depression and anxiety?”* 52 yo woman *“Can Black Cohosh cause a skin rash”*
Balancing risk vs. benefit to either use or withdraw	26 (11.4%) 58 yo woman *“I have hot flushes, and mood swings. My doctor pushed me to use HRT but I refuse to take it. We have a right to choose an alternative. Can you tell me the risks and benefits of HRT?”*	26 (37.1%) 58 yo woman *“I have been on Kliogest (estradiol/ norethisterone) for 2 years for hot flushes and for my bones. I’ve just read that HRT can cause harm but it really helps me. Should I stay on my HRT or not? Can you help?”*	19 (3.5%) 61 yo woman *”What are the risks and benefits of Livial® (tibolone) used long term?”*
Safety concern – seeking to change to an alternative product	1 (0.4%) 48 yo woman Treated for thyroid carcinoma; GP believes this caused early menopause. Tried HRT but found side effects intolerable; lowering the dose didn’t help *“.. I was then changed to Vagifem (estradiol) pessaries but am now experiencing vaginal bleeding. Is this normal? What can I try next?”*	12 (17.1%) 67 yo woman *“I’ve been on HRT for 15 years for osteoporosis and my ‘heart’. I read that my drug can cause cancer. Would herbal HRT or a patch be safer and worth a try instead? “*	42 (7.7%) 50 yo woman “*Could swapping from Kliogest (estradiol* + *norethisterone) to Premia® (conjugated estrogens* + *medroxyprogesterone) ease headaches and breakthrough bleeding?”*
Reassurance to use or to use with other drugs or comorbidities	41 (17.9%) 65 yo woman *“I have depression, will taking estrogen help?”* 61 yo woman *“I used HRT since 1987 and it helps but now have been diagnosed with anxiety. I want to try HRT patches instead? Is this OK to use?*	1 (1.4%) 62 yo woman *“Can I use HRT with my thyroid medicines?”*	138 (25.2%) 58 yo woman *“What is the interaction between Estradot® (estradiol) and raloxifene?”* 47 yo woman *“Do I need both estrogen and progesterone to regulate estrogen levels?”*
2. Pragmatics of use	n = 41 (17.9%)	Nil	n = 72 (13.2%)
General information	23 (10.0%) 52 yo woman *“Can you give me more information on HRT?”*	Nil	18 (3.2%) 52 yo woman *“Can you give me some information on Femoston® (estradiol* + *dydrogesterone)?”*
Logistics	7 (3.1%) 52 yo woman *“I used HRT gel in the UK and it was great, is there any HRT gel in Australia?”*	Nil	34 (6.2%) 65 yo woman *“Is there a generic for Livial® (tibolone)?”*
Seeking a strategy to use	11 (4.8%) 48 yo woman *'Regarding HRT, do Premarin® (conjugated estrogens) and Provera® (medroxy-progesterone) have to be taken together?*	Nil	20 (3.7%) 85 yo woman *“How do I use Ovestin® (estriol) pessaries?”*
3. Therapy options	n = 32 (14.0%)	n = 1 (1.4%)	n = 83 (15.1%)
Efficacy considerations	20 (8.7%) 50 years old male Wife on Promensil and it helped her hot flushes *Have you heard how wonderful Promensil® (red clover) is for menopause symptoms?”*	Nil	39 (7.1%) 42 yo woman *“Is Kliovance® (estradiol* + *norethisterone) ok to reduce flow?”* 62 yo woman *Are chasteberry and safe effective in treating menopausal symptoms?*
Symptom management	11 (4.8%) 47 yo woman *“My mother and sister had osteoporosis. How can I prevent osteoporosis?”*	Nil	18 (3.3%) 70 yo woman *“What can I use to improve my libido?”*
Dissatisfaction with previous treatment—either to improve effectiveness or as experiencing side effects	1 (0.4%) 58 yo woman had a hysterectomy; previously tried Premarin*®* (conjugated estrogens) but it caused dizziness. She now takes Menorest (estradiol) but has now developed sore “Can I use Remifemin® (black cohosh) as an alternative to HRT?”**	1 (1.4%) 75 yo woman *“Can I use Fosamax® (alendronic acid)as an alternative to hormone replacement therapy?”*	5 (0.9%) 63 yo woman *“What are the options available for estrogen replacement therapy if estradiol 2 mg per day isn’t working?”*
Availability	Nil	Nil	21 (3.8%) 63 yo woman *“Ogen® (estropipate) has been discontinued. So what alternative medicines to Ogen® are there?”* 55 yo woman *“What is the closest estrogen form that I can use if estropipate is taken off the market?”*
4. Regimen comparison	n = 20 (8.7%)	Nil	n = 35 (6.4%)
Comparison	72 yo women had hysterectomy and had been on HRT for 27 years. Doctor changed HRT from E*s*tigyn 0.01mcg to Ogen 0.625 mg. She became nauseous an hour after taking Ogen *“Is my Ogen® (estropipate) dose comparable to Estigyn® (estradiol)?”*	Nil	53 yo woman “*Is Livial® (tibolone) safer than Progynova® (estradiol) for me?”*

Sometimes the cancer fear was transferred to other symptoms or concerns. Following his wife’s death from heart failure, a 60-year-old male was concerned that HT might have been the underlying cause (Table 3). Breast cancer was mentioned at other time periods; it was not a fear of the disease per se but to decide whether they could (or should) use HT or an alternative strategy.

Women asking for help to balance the risks and benefits to either use or withdraw HT, based on their individual circumstances, was a particular feature immediately after WHI (*Time 2a);* while before WHI and later, there was a focus on clarifying the cause of a particular symptom in relation to HT. Before and later after WHI women more commonly sought reassurance to use HT with other medicines or in the presence of other diseases.

*Pragmatics of use* ranked highly in *Times 1* and *3*, where changes in medicines access, particularly in later years, prompted a need for decision-making. Similarly, calls about *therapy options* and *regimen comparisons* at *Times 1* and *3* were not evident at *Time 2a*. Over time, the nature of HT safety questions evolved to include whether vaginal HT produced similar effects to systemic HT (oral or transdermal) and calls asking for alternative forms of treatment. For non-hormone therapy, women asked whether HM, antidepressants, or bisphosphonates were effective in managing menopause. Women older than 60 years sought reassurance to use HT across the period of our study (1996–2010) despite guidelines after 2002 deeming HT unsuitable for this cohort [38] (Table 3).

**Fig. 2:**
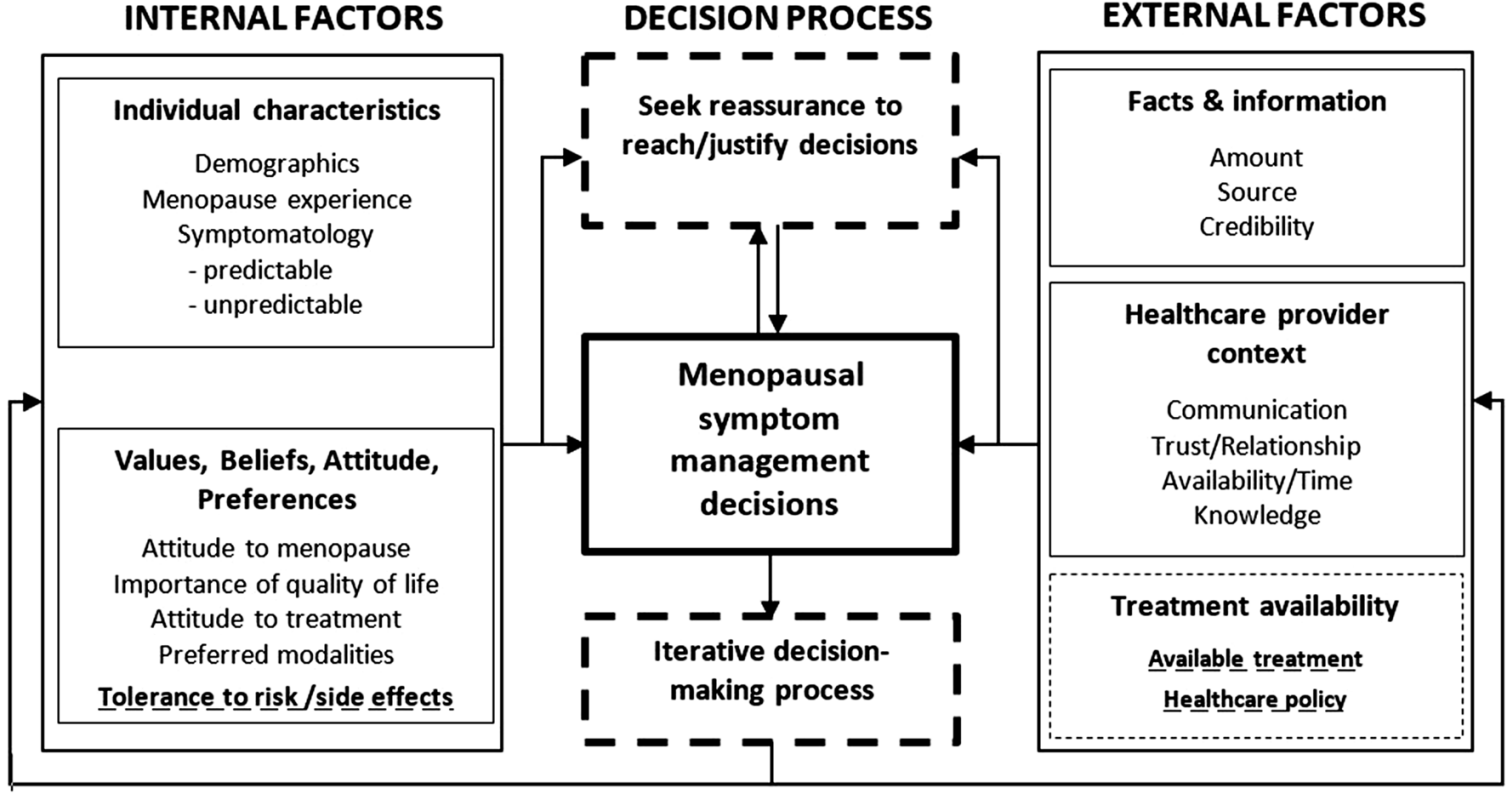
Decision-making model in menopause management. New factors or processes observed in this study when compared to previous decision-making models [19, 20] are in bold dashed-line and dashed-line boxes

Our data demonstrated the complex and dynamic interplay of internal and external factors that influence women who seek information for reassurance, to reach or justify a decision about managing menopause symptoms (Fig. 2). Information-seeking occurred in response to heightened uncertainty: worrying symptoms, therapy dissatisfaction, inadequate or conflicting information, perceived risk, or change to treatment availability. Any ambiguity stimulated decision reassessment. The more intense the uncertainty (e.g. negative media about HT), the more women relied on emotions to emphasise risk over benefit. Selected vignettes highlight the iterative nature of this decision-making (Additional file 2).

## Discussion

WHI 2002 [5] was the ‘top medical story of 2002’, creating the ‘HT scare’ [9]. Negative media was the main driver of information-seeking for decision-making; calls increased 400% in the first two weeks and remained high for the subsequent two months. Maladaptive risk perception is a cornerstone of cognitive models of decision making under risk and uncertainty, where worried individuals generally overestimate negative outcomes [39], and emotional reactions to perceived risk often diverge from cognitive assessments of those risks, driving behaviours such as help-seeking [40]. While the effect on help-seeking was relatively short-lived, consumer concerns about safety persisted for years after WHI 2002. Despite subsequent media reports of HT benefits [9] in specified cohorts [11], there were few calls prompted by media reports after 2006. In an invited review about media and the consumer, Shapiro highlighted the pervasive impact of media on public perceptions of risk, citing Vincent Covello (Center for Risk Communication, Columbia University): *“research has shown that strong beliefs about risk, once formed, change very slowly and are extraordinarily persistent in the face of contrary evidence.’’ *[41]

The subsequent HT-favourable findings of cardiovascular benefit in selected cohorts [11] did not elicit the same level of public attention as WHI 2002. Women’s perception may have been influenced by other information sources (e.g. peers and clinicians) [9] or may reflect persistent concern about breast cancer [11]. Our findings support the notion that the fear of cancer overshadowed any beneficial effect of HT on cardiovascular health [42].

There were proportionally fewer menopause-related calls over time. While this might simply reflect lower HT use due to progressive product withdrawals after 2002, few studies assessing the relationship between product withdrawal and use could be retrieved [42–44]. In addition, distrust in medical science, given the ‘pendulum swings’ in HT evidence [21], potentially left women feeling they had no alternative but to bear with their symptoms without pharmacological intervention [27].

We found that women considering menopause therapy sought reassurance about their decision-making before and after WHI 2002, but their priorities differed. Afterwards, women were more likely to seek confirmation that HT or HM were safe to use with their other medicines, suggesting increased interest in HM as a substitute for HT [24, 27]. The potential for interactions with over-the-counter medicines such as HM is a concern for consumers who self-medicate without consulting their clinician [45]. Women with severe menopausal symptoms might also experience other symptoms [2], or use more healthcare services [4], which may mean use of medicines other than HT. There may have also been a change in the treatment objective. Before WHI 2002 some clinicians prescribed HT for women who were symptom free; while afterwards HT use was largely limited to patient requests for symptom relief [21].

We found that changes in treatment access or availability influenced information-seeking behaviour and prompted women to seek substitute treatment. After WHI 2002, women requested information on substitute treatments when HT products were progressively removed from government subsidy and subsequently the Australian market. Calls about HM primarily originated from areas with good access to service centres where a pharmacy, health store, or naturopath are more likely to be located. Our finding is consistent with a survey of HM use by 2,020 Australian middle-aged women which found a higher prevalence of HM use for menopausal symptoms among women residing in metropolitan areas [46] perhaps reflecting increased availability and income.

To our knowledge, no other study has examined women’s information-seeking and decision-making about managing menopause before and for more than seven years after WHI 2002. These data reflect the real-world issues faced by women and the significant impact of negative media reporting. The study’s longitudinal nature also allowed us to examine any persisting impact of WHI 2002.

We acknowledge some study limitations. The data were collected as part of routine MCC activity so there was no opportunity to prospectively collect additional data of interest. The MCCs were in Australia, so our results might not reflect the concerns of women in other places with different cultures and ethnicities. Indications for medicines were not captured, so we were unable to explore concerns about the use of non-hormone therapy for menopause [17]. The two data sources might not be fully comparable given the differences in coverage and time frames. Our study period ended in 2010, prior to the Global Consensus Statement on Menopausal Hormone Therapy in 2013 [47]—the official reassurance made by various medical societies about the safe use of HT in specific cohorts of women. We need to know more about women’s information needs for all available treatments, especially after 2013.

Building on previous models of decision-making in menopause [19, 20], we identified that women information-seek for reassurance from a trusted source, to assist or justify a decision [48]. This is influenced by heightened intolerance to risk, associated with a change in competing internal factors (woman’s specific symptom concerns and tolerance to risk), and external factors (the impact of negative or conflicting information, source trust and product availability (Fig. 2). Decision-making is an iterative process. Accessing HT or other therapy involves a discourse between a woman and her clinician but we observed that women sought information or reassurance to support a new decision based on dynamic changes in internal (e.g. experiencing a side effect or new symptoms) and external factors (including conflicting medical advice) about how she will ultimately manage her symptoms. Negative or conflicting source information, worrying symptoms, perceived treatment risk, and decreased treatment availability contribute to perceived risk predominance. If other factors emerge that change the dynamics of the decision-making process, the iterative cycle will recommence (Fig. 2).

## Conclusions

Negative WHI 2002 media reports escalated information-seeking for decision-making about managing menopausal symptoms. While safety concerns and over-estimation of risk persisted, the factors influencing the nature and intensity of information-seeking changed over time and over the woman’s menopause journey, requiring reconsideration of previous decisions. Reassurance to reach or justify decisions from a perceived trusted source can support informed and shared decision-making.

## Supplementary Information

**Additional file 1**. Call characteristics comparison. **Table 1.** Comparison of call characteristics between menopause-related and the rest of calls. **Table 2.** Comparison of caller characteristics between Queensland and rest of Australia.

**Additional file 2**. Three call vignettes.

## Data Availability

The data that support the findings of this study are available from Mater Pharmacy, Mater Health, SEQ and NPS MedicineWise but restrictions apply to the availability of these data, which were used under license for the current study, and so are not publicly available. Data are however available from the authors upon reasonable request and with permission of Mater Pharmacy, Mater Health, SEQ and NPS MedicineWise.

## Abbreviations

EPT: Estrogen plus progestogen therapy
ET: Estrogen therapy
HM: Herbal medicines
HT: Hormone therapy
MCC: Medicines call centre
NPS: National prescribing service
ML: National prescribing service medicines line
QMH: Queensland medication helpline
RECORD: REporting of studies conducted using observational routinely-collected health data (guideline)
ROC: Rest of calls
VMS: Vasomotor symptoms
WHI: Women’s health initiative
