# Supplementary material for: Long term impact of the WHI studies on information-seeking and decision-making in menopause symptoms management: a longitudinal analysis of questions to a medicines call centre
Source: BMC Womens Health. 2021 Oct 4;21:348. doi: 10.1186/s12905-021-01478-z (PMC8491426; doi:10.1186/s12905-021-01478-z)
Supplement: Supplementary file 1 — Additional file 1. Call characteristics comparison. Table 1. Comparison of call characteristics between menopause-related and the rest of calls. Table 2. Comparison of caller characteristics between Queensland and rest of Australia. [file 12905_2021_1478_MOESM1_ESM.docx]

**Appendix A Call characteristics comparison**

1. Comparison between menopause-related and rest of calls

Table 1 compares characteristics of menopause-related versus rest of calls (ROC). Key characteristics between menopause-related and ROC differed (notably callers’ gender and age, patients’ age, and relationship), however, these characteristics are relatively stable over time in ROC.

Table 1 Comparison of call characteristics between menopause-related and the rest of calls

| **Characteristics** | **Queensland Medication Helpline** | | | | **NPS *Medicines Line*** | |
| --- | --- | --- | --- | --- | --- | --- |
|  | ***Time 1*** | | ***Time 2*** | | ***Time 3*** | |
|  | **Menopause**  **(n = 742)** | **Rest of calls**  **(n = 17,321)** | **Menopause**  **(n = 126)** | **Rest of calls**  **(n = 633)** | **Menopause**  **(n = 961)** | **Rest of calls**  **(n = 122,264)** |
| **Callers age, years** | | | | | | |
| Mean (SD) | 56.5 (9.6) | 51.2 (17.2) | 59.3 (8.5) | 52.1 (16.4) | 57.7 (10.5) | 50.3 (17.6) |
| **Callers gender, %** | | | | | | |
| Male | 4.0 | 24.6 | 4.0 | 17.5 | 3.2 | 23.2 |
| Female | 94.2 | 74.2 | 95.2 | 79.8 | 96.8 | 76.6 |
| **Patients age, years** |  |  |  |  |  |  |
| Mean (SD) | 57.1 (9.7) | 44.1 (24.8) | 59.9 (8.9) | 44.5 (26.3) | 58.0 (10.2) | 46.8 (23.8) |
| **Relationship of caller, %** | | | | | | |
| Self | 89.1 | 70.4 | 88.9 | 64.5 | 95.1 | 71.2 |
| Partner | 2.2 | 5.1 | 4.0 | 7.1 | 1.9 | 5.7 |
| Client (carer/patient) | 2.0 | 4.5 | - | 3.9 | 1.7 | 3.2 |
| Child/friend/other family | 2.8 | 15.1 | 1.6 | 11.7 | 0.8 | 16.7 |
| Parent | 1.2 | 2.2 | 0.8 | 3.5 | 0.5 | 2.9 |
| **Call motivation, %** | | | | | | |
| Inadequate information | 14.2 | 17.8 | 9.5 | 27.5 | 38.5 | 46.7 |
| Second opinion | 14.7 | 12.5 | 8.7 | 22.4 | 24.5 | 23.5 |
| Worrying symptom | 22.2 | 20.5 | 4.8 | 19.3 | 19.7 | 17.8 |
| Conflicting information | 7.8 | 6.8 | 0.8 | 6.6 | 8.8 | 5.6 |
| Other | 13.5 | 15.7 | 1.6 | 6.6 | 6.8 | 5.8 |
| Media | 7.4 | 5.2 | 66.7 | 5.7 | 1.3 | 0.5 |
| **Enquiry Type, %^a^** | | | | | | |
| Side effects* | 24.8 | 24.3 | 15.9 | 21.6 | 23.2 | 19.6 |
| Pragmatics of use | 16.2 | 18.0 | 14.1 | 16.1 | 11.8 | 14.0 |
| Treatment/prophylaxis | 13.4 | 11.7 | 8.2 | 11.8 | 8.5 | 10.2 |
| Risk/benefit* | 12.7 | 10.5 | 46.5 | 17.1 | 17.4 | 15.7 |
| Logistics and miscellaneous | 12.7 | 12.3 | 8.2 | 11.8 | 9.4 | 12.9 |
| Mechanism/profile | 12.5 | 12.7 | 4.7 | 8.1 | 14.4 | 12.1 |
| Interaction* | 7.6 | 10.5 | 2.4 | 13.5 | 15.1 | 15.2 |
| *Enquiry Type – Safety** | *45.1* | *45.3* | *64.8* | *52.2* | *55.7* | *50.5* |
| **ARIA index, relative call frequency^b^** | | | | | | |
| Highly accessible | 1.14 | 1.10 | 1.17 | 1.07 | 1.22 | 1.24 |
| Accessible | 0.55 | 0.68 | 0.46 | 0.77 | 0.48 | 0.46 |
| Moderately accessible | 0.48 | 0.49 | 0.44 | 0.43 | 0.32 | 0.32 |
| Remote | 0.42 | 0.41 | - | 0.44 | 0.53 | 0.66 |
| Very remote | 0.56 | 0.44 | 0.80 | 0.56 | 1.00 | 0.71 |

NPS = National Prescribing Service, *Time 1* = pre-the Women’s Health Initiative (WHI) 2002 study (1996 – 9 July 2002), *Time 2* = post-WHI 2002 (10 July – 19 September 2002), *Time 3 =* September 2002 – June 2010, SD = standard deviation, ARIA = Accessibility Remoteness Index of Australia

a Some cases in Queensland Medication Helpline have more than one question. Proportion in this variable reflects the proportion of the number of enquiry types to total enquiry type (*Time 1*, number (n) menopause questions = 1,739, n rest of calls = 33,652; *Time 2*, n menopause = 170, n rest of calls = 849)

b Relative call frequency was calculated by dividing the proportion of calls by proportion of population in the specific ARIA index

1. Comparison between Queensland and other states in the *Medicines Line* and between *Queensland Medication* *Helpline* and *Medicines Line*

Queensland Medication Helpline (QMH) data was only from Queensland consumers so we assessed whether call characteristics differed between Queensland and other states in the Medicines Line (ML) dataset and between Queensland callers of QMH and ML.

There were no differences in caller gender, relationship between caller and patient, accessibility to service centres across geographical locations (Queensland versus other states/territories) or over time (pre- versus post-WHI). However, the mean ages of callers and patients in the rest of Australia post-WHI 2002 were approximately 2 years younger than in Queensland. This can be explained by differences in funder marketing strategies between the state-wide and national services. QMH had limited funding so marketing was opportunistic*,* including radio talk-back which mostly reached an older audience; while ML used multiple media for marketing, including the internet, which can reach a wider audience.

The enquiry profile did not differ: the frequency for the highest-ranked enquiry type (side-effects) was similar for Queensland callers pre- and post- WHI 2002 (*Times 1* and *3*). While other common enquiry types differed between *Times 1* and *3*, the similarities of *Time 3* callers from Queensland and other states and territories suggests that enquiry types typically reflect this later time frame more than the caller location.

One in five callers were motivated by worrying symptoms and one in 12 Queensland callers were motivated to call because of conflicting information (*Time* 1, 7.8%; *Time 3*, 8.1%). The number of calls prompted by inadequate information was higher from Queensland callers in *Time 3* than in *Time 1.* The similarity between *Time 3* callers from Queensland versus all other states and territories again suggests that the motivation to call more likely reflects the fact that consumers have progressively become more proactive help-seekers over time, irrespective of their location.

Table 2 Comparison of caller characteristics between Queensland and rest of Australia

| **Characteristics** | **QMH**  ***Time 1***  ***(n = 742)*** | ***ML*** | | **p-value** | **Note** |
| --- | --- | --- | --- | --- | --- |
|  |  | ***Time 3 QLD***  **(n = 407)** | ***Time 3 RoA***  **(n = 549)** |  |  |
| **Callers age, years** | | | | <0.001 | **Comparison, ΔMean (95% CI)** |
| Mean (SD) | 56.5 (9.6) | 58.8 (10.6) | 56.9 (10.4) |  | T1 vs. T3 QLD: -2.30 (-3.76, -0.84) |
|  |  |  |  |  | T3 QLD vs. T3 RoA: 1.90 (0.35, 3.45) |
| **Callers gender, %** | | | | 0.522 |  |
| Male | 4.0 | 3.7 | 2.9 |  |  |
| Female | 94.2 | 96.3 | 97.1 |  |  |
| **Patients age, years** | | | | 0.006 | **Comparison, ΔMean (95% CI)** |
| Mean (SD) | 57.1 (9.7) | 59.0 (10.6) | 57.3 (10.0) |  | T1 vs. T3 QLD: -1.90 (-3.35, -0.45) |
|  |  |  |  |  | T3 QLD vs. T3 RoA: 1.70 (0.16, 3.24) |
| **Relationship of caller, %** | | | | 0.107 |  |
| Self | 89.1 | 95.6 | 94.7 |  |  |
| Partner | 2.2 | 2.2 | 1.6 |  |  |
| Client (carer/patient) | 2.0 | 1.5 | 1.8 |  |  |
| Child/friend/other family | 2.8 | 0.2 | 1.3 |  |  |
| Parent | 1.2 | 0.5 | 0.5 |  |  |
| **Call motivation, %** | | | | <0.001 |  |
| Inadequate information^a^ | 14.2 | 38.3 | 38.6 |  |  |
| Second opinion | 14.7 | 22.6 | 25.7 |  |  |
| Worrying symptom | 22.2 | 20.1 | 19.5 |  |  |
| Conflicting information | 7.8 | 8.1 | 9.5 |  |  |
| Other^a^ | 13.5 | 8.6 | 5.3 |  |  |
| Media^a^ | 7.4 | 1.7 | 1.1 |  |  |
| **Enquiry Type, %**^b^ | | | | <0.001 |  |
| Side effects | 24.8 | 21.9 | 24.0 |  |  |
| Pragmatics of use^a^ | 16.2 | 8.4 | 13.7 |  |  |
| Treatment/prophylaxis | 13.4 | 9.1 | 8.2 |  |  |
| Risk/benefit | 12.7 | 17.7 | 17.3 |  |  |
| Logistics and miscellaneous | 12.7 | 6.9 | 11.1 |  |  |
| Mechanism/profile^a^ | 12.5 | 18.7 | 11.7 |  |  |
| Interaction^a^ | 7.6 | 16.7 | 14.0 |  |  |
| **ARIA index, relative call frequency**^c, d^ | | | | <0.001 |  |
| Highly accessible | 1.14 | 1.22 | 1.23 |  |  |
| Accessible | 0.55 | 0.49 | 0.46 |  |  |
| Moderately accessible | 0.48 | 0.30 | 0.34 |  |  |
| Remote | 0.42 | 0.49 | 0.36 |  |  |
| Very remote | 0.56 | 0.74 | 0.73 |  |  |

QMH = Queensland Medication Helpline, ML = National Prescribing Service Medicines Line, *Time 1* = calls to QMH prior to the Women’s Health Initiative (WHI) 2002, *Time 3 QLD* = calls to ML originating from the state of Queensland, *Time 3 RoA* = calls to ML originating from all Australia states/territories except Queensland, SD = standard deviation, ΔMean = mean difference between two groups, CI = confidence interval, ARIA = Accessibility Remoteness Index of Australia

Comparison between group in callers and patients age were obtained from Tukey’s post-hoc analysis. Only comparison with statistically significant result was presented

a Denotes the category whose column proportion of some categories differ significantly to others at the p=0.05 with Bonferroni correction

b Some cases in Queensland Medication Helpline have more than one question. Proportion in this variable reflects the proportion of the number of enquiry types to total enquiry type (*Time 1*, number of menopause-related questions = 1,739)

c Relative call frequency was calculated by dividing the proportion of calls by the proportion of population in the specific ARIA index

d We combined the “Remote” and “Very remote” categories in the Pearson’s chi-square test to meet the assumption of the test (i.e. less than 20% of cells has expected value less than 5)
