# Supplementary material for: Long term impact of the WHI studies on information-seeking and decision-making in menopause symptoms management: a longitudinal analysis of questions to a medicines call centre
Source: BMC Womens Health. 2021 Oct 4;21:348. doi: 10.1186/s12905-021-01478-z (PMC8491426; doi:10.1186/s12905-021-01478-z)
Supplement: Supplementary file 2 — Additional file 2. Three call vignettes. [file 12905_2021_1478_MOESM2_ESM.docx]

**Appendix B: Call vignettes**

**Vignette 1: menopausal symptoms from abrupt withdrawal**

48-year-old woman had spinal fusion surgery at 39-year-old and has never had a menstrual period after that. She has continued to suffer from night sweats on and off for the last nine years.

She also takes mirtazapine 45 mg at night for depression, *Hypnodorm*^®^ (flunitrazepam) 1 mg at night for insomnia and *Panadeine Forte*^®^ (paracetamol 500 mg/codeine 30 mg) 1-2 tablets when necessary for pain (she takes 6-8 tablets daily).

Over the years she has tried every type of hormone replacement therapy (HRT) – nasal, gels, patches, compounded progesterone, tibolone, even herbal medicines – but nothing really helped. Every doctor she has consulted has a different opinion as to why this is happening, but they just offer another form of HRT rather than helping her deal with her symptoms.

Last week, she got sick of the vicious cycle and decided to throw her current HRT (*Angeliq^®^* i.e. estradiol 1 mg/drospirenone 2 mg) in the bin. She is now experiencing severe headaches, worsening night sweats and anxiety – she can’t settle or sleep.

*“Please help me - It’s getting worse – I can’t think anymore. Should I go back on HRT again or not? – Will it make things better or worse?”*

**Vignette 2: Conflicting advice causing anxiety**

In the following case, it is possible to see the effects of conflicting advice from her specialist and her general practitioner (GP). This has created considerable uncertainty and anxiety as to what to do.

67-year-old woman has been taking HRT since she was 51-year-old and is currently taking *Ogen*^®^ (piperazine estrone sulfate) 1.25 mg and *Provera*^®^ (medroxyprogesterone acetate) 5 mg. She also takes *Tofranil*^®^ (imipramine) 100 mg at night for sleep and anxiety.

*“I have been told to fine down my HRT dose by my specialist with a view to cutting to zero over time – but my GP said to stay on it as I have had no problems over the last 16 years. Who should I believe?” I’m now worried about how safe it is long term. Should I cut down my dose?*

**Vignette 3: A hormone replacement therapy journey**

The final case study clearly shows the treatment of menopausal symptoms as a journey involving the trial of a range of herbal medicines and HRT. It also highlights the role a lack of information or inadequate information plays in creating anxiety or dissonance leading to information-seeking from an alternative source.

49-year-old woman is perimenopausal and symptoms – hot flushes, worse at night - began about 3 years ago. She has *“aversion to synthetic drugs and is sensitive to drugs".* She was trying to get through with acupuncture, black cohosh, red clover and Chinese medicines, until she started getting severe headaches. She finally decided to go to her GP who put her on *Premia^®^ 2.5 continuous* (conjugated equine estrogens 0.625 mg / medroxyprogesterone acetate 2.5 mg) which fixed her headache immediately. Now he's put her on *Premarin^®^* (conjugated equine estrogens) 6.25 mg as well until her symptoms improve. *“He said this was better for me but didn’t tell me why.”*

*“Now I have started getting a bitter metallic taste which doesn’t improve with meditation, food or anything. Is my new HRT causing the metallic taste? Maybe my body is reacting while adjusting to the medicine? I’m getting really anxious - what should I do?”*
